# Maternal undernutrition aggravates renal tubular necrosis and interstitial fibrosis after unilateral ureteral obstruction in male rat offspring

**DOI:** 10.1371/journal.pone.0221686

**Published:** 2019-09-03

**Authors:** Midori Awazu, Tokiya Abe, Akinori Hashiguchi, Mariko Hida

**Affiliations:** 1 Department of Pediatrics, Keio University School of Medicine, Tokyo, Japan; 2 Department of Pathology, Keio University School of Medicine, Tokyo, Japan; Max Delbruck Centrum fur Molekulare Medizin Berlin Buch, GERMANY

## Abstract

Maternal undernutrition is known to reduce glomerular number but it may also affect tubulointerstitium, capillary density, and response to oxidative stress. To investigate whether the latter elements are affected, we examined the response to unilateral ureteral obstruction (UUO), an established model of renal tubulointerstitial fibrosis, in the kidney of offspring from control and nutrient restricted rats. Six-week old male offspring from rats given food ad libitum (CON) and those subjected to 50% food restriction throughout pregnancy (NR) were subjected to UUO for 7 days. Body weight was significantly lower in NR. Systolic blood pressure and blood urea nitrogen increased similarly in CON and NR after UUO. Tubular necrosis in the obstructed kidney, on the other hand, was more extensive in NR. Also, the collagen area, a marker of fibrosis, of the obstructed kidney was significantly increased compared with the contralateral kidney only in NR. Capillary density was decreased similarly in the obstructed kidney of CON and NR compared with the contralateral kidney. Urine nitrate/nitrite, a marker of nitric oxide production, from the obstructed kidney was significantly increased in NR compared with CON. Nitrotyrosine, a marker of nitric oxide-mediated free radical injury, was increased in the obstructed kidney compared with the contralateral kidney in both CON and NR, but the extent was significantly greater in NR.

In conclusion, more severe tubular necrosis and fibrosis after UUO was observed in NR, which was thought to be due to increased nitrosative stress.

## Introduction

The concept of developmental origins of health and disease, also referred to as programming, is important in the pathogenesis of kidney diseases [1]. Low birth weight is associated with a risk for renal diseases as well as hypertension, and reduced glomerular number is linked to these associations epidemiologically as well as experimentally [1]. Disturbed intrauterine environment, however, may also affect tubules, interstitium, capillary density, and oxidative stress [2, 3]. The expression of tubular transporters has been shown to be altered by maternal low protein diet [4]. Subjects with extremely low birth weight were reported to have various tubular dysfunctions [2]. Not much attention has been paid to the effect of an adverse intrauterine environment on the interstitium, despite the fact that interstitial fibrosis is an important factor in the progression of kidney disease. Vehaskari et al. [5] reported that the offspring of rats fed a low protein diet showed normal renal histology at 8 weeks of age. At 18 months, however, low protein group showed more pronounced tubular atrophy/dilation and interstitial fibrosis along with more severe glomerulosclerosis compared with controls. Others reported, on the other hand, that renal histology including glomerulosclerosis and interstitial fibrosis was not different at 32 and 100 weeks between female offspring of protein-restricted rats and controls, although renal function was deteriorated in the former at 100 weeks [6]. While aging may or may not reveal the effect of an adverse intrauterine environment, the insult may become apparent by a more severe secondary injury. In support of this possibility, a previous study demonstrated that renal tubular injury score was similar to controls at baseline in rats with intrauterine growth restriction (IUGR) induced by reduced uterine perfusion [7]. After ischemia-reperfusion, however, histological tubular injury was significantly more severe in IUGR rats. Tubulointerstitial fibrosis after injection of anti-Thy-1 antibody was also reported to be increased in IUGR rats induced by uterine artery ligation [8]. In these IUGR rats, however, glomeruli were more sclerosed compared with controls, which may explain the more pronounced fibrosis *via* decreased peritubular capillary blood flow. In humans, low birth weight was suggested to be a risk factor for the development of renal scarring after urinary tract infection [9]. This suggests that an adverse intrauterine environment affects the development of tubulointerstitial injury directly. Experimental studies that examined the primary injury to the interstitium in IUGR animals are lacking.

In the present study, therefore, we examined the response to unilateral ureteral obstruction (UUO), an established model of renal tubulointerstitial fibrosis, in the male offspring from control and nutrient restricted rat dams.

## Materials and methods

### Experimental animals

Approval was obtained from the Animal Experiment Committee of Keio University. Female Sprague Dawley rats at day 1 of pregnancy were purchased from Sankyo Labo Service corporation, Inc. (Tokyo, Japan). Animal experiments were performed at the laboratory of department of Pediatrics, Keio University School of Medicine. The male offspring of dams given food *ad libitum* (n = 8 litters, 1 offspring/litter) and those subjected to 50% food restriction throughout pregnancy (n = 7 litters, 1 offspring/litter) were examined. Sample size was determined according to the review for DOHaD research [10] and our previous study [11]. We previously showed that maternal nutrient restriction suppressed ureteric branching during the fetal period and induced low glomerular number in adult kidneys. Male rats were examined because the effect of programming is stronger in males than females in general [12]. Also most of our current knowledge of UUO has been obtained from studies using male rats. At 6 weeks of age, rats were anesthetized with an intraperitoneal injection of sodium pentobarbital and blood pressure was measured by a tail cuff method using BP-98A (Softron, Japan). Briefly, rats were kept at 36°C and blood pressure measurements were made for at least 5 times within a few minutes. High initial values and or the maximum and the minimum values were discarded and the remaining values were averaged. Blood was collected from the tail vein. They then underwent left unilateral ureteral ligation through an abdominal midline incision [13]. After 7 days, rats were anesthetized and blood pressure was measured in the same way. Blood were collected from the tail vein and after abdominal incision, urine was obtained from the obstructed kidney by pelvic puncture. Kidneys were then removed and fixed in 4% paraformaldehyde for histological examination or lysed for immunoblot analysis. Although comparison between obstructed and sham-operated kidney is ideal, we used contralateral kidney as a control because a previous study reported no difference between sham-operated kidney and contralateral kidney regarding apoptosis and fibrosis [14].

### Reagents

The following primary antibodies were used: CD31 (clone TLD-3A12, BioRad, Helcules, CA), eNOS (BD Transduction Laboratories, San Diego, CA), nitrotyrosine (Calbiochem, San Diego, CA), and GAPDH (Cell Signaling Technology, Beverly, MA). Horseradish peroxidase-conjugated anti-mouse IgG and anti-rabbit IgG were from Amersham (Buckinghamshire, UK).

### Histology

After fixation, kidneys were embedded in paraffin. For quantification of fibrosis, specimens were stained with elastic van Gieson (EVG). A whole slide image (WSI) of each specimen was acquired using the NanoZoomer 2.0HT (Hamamatsu Photonics K.K., Hamamatsu, Japan). The WSI pixels were classified into collagen fibers, elastic fibers, nucleus, cytoplasm, and non-tissue component as previously reported [15]. At least 30 fields of view were examined in the cortex excluding the necrotic area. For the assessment of acute tubular necrosis (ATN), sections were stained with hematoxylin and eosin.

### Immunohistochemistry

Sections were deparaffinized and rehydrated. Staining was performed with a Leica Biosystems' Bond-Max (Leica Biosystems, Melbourne, Australia). Epitope retrieval was performed by incubating the slides in Bond Epitope Retrieval Solution 1 (Leica Microsystems) at 100°C for 20 min. The dilution of anti-CD31 antibody was 1:20000. Sections were photographed at a magnification of x200 and CD31-positive area was measured using ImageJ software (National Institute of Health, Bethesda, MD).

### Immunoblot analysis

Kidneys were lysed in solubilization buffer containing 20 mM HEPES (pH 7.2), 1% Triton X-100, 10% glycerol, 20 mM sodium fluoride, 1 mM sodium orthovanadate, 1 mM PMSF, 10 μg/mL aprotinin, and 10 μg/mL leupeptin. Insoluble material was removed by centrifugation (10,500 *g*, 10 min). Lysates were resolved by SDS-PAGE and transferred to PVDF membranes (Immobilon, Millipore, Bedford, MA). Nonspecific binding sites were blocked in TBS buffer (10 mM Tris•HCl, pH 7.4, 0.15 M NaCl) containing 0.1% Tween 20 and 5% BSA overnight at 4ºC or for 1 h at 25°C. Antibodies were added to TBS containing 0.1% Tween 20 with 5% BSA and incubated with mixing for 24 h at 4°C. After incubating with secondary antibody, bound antibodies were detected using the ECL Western blotting system (Amersham, Arlington Heights, IL) and visualized using Chemi Doc system (Bio-Rad Laboratories, Hercules, CA). All experiments were repeated at least three times on separate samples.

### Urine nitrate/nitrite (NOx)

*Urine* was stored at -80°C until the time of assay. Urine NOx, metabolites of nitric oxide (NO), was measured using Nitric Oxide (total), detection kit (Enzo Life Sciences, Farmingdale, NY). Briefly, 50 μl of samples and standards were placed into duplicate wells, followed by addition of 25 μl of NADH and 25 μl of nitrate reductase. The plate was then incubated for 30 min at 37°C. Then 50 μl of the Griess reagent I and II were placed into each well. The optical density of each well was read at 540 nm after blanking against the blank wells.

### Serum and urine chemistry measurements

Blood urea nitrogen was measured using Fuji Dry Chem NX500V (Fujifilm, Tokyo, Japan). Urine chemistry measurements were made at SRL Inc (Tokyo, Japan). Urine osmolality, protein, sodium, N-acetyl-β-D-glucosaminidase, magnesium, and creatinine were measured by freezing point depression method, pyrogallol red method, electrode method, colorimetric method, xylidyl blue method, and enzymatic method, respectively. Fractional excretion of magnesium was reported to correlate with tubulointerstitial fibrosis in human nephrotic syndrome [16]. Since fractional excretion values are influenced by glomerular filtration rate, we used urine magnesium corrected by creatinine.

### Statistical analysis

The results are expressed as means±SE. After checking normal distribution of the data, statistical analysis was performed with paired *t* test, unpaired *t* test, ANOVA followed by Tukey’s test as appropriate using JMP software (SAS institute Inc., NC). Statistical significance was determined as *P* < 0.05.

## Results

### Body weight, blood pressure, and blood urea nitrogen

Body weight of offspring from dams subjected to 50% food restriction throughout pregnancy (NR) was significantly lower than that of offspring from dams given food ad libitum (CON)(Table 1). Systolic pressure increased significantly after UUO in both CON and NR, which may be due to the growth rather than the effect of UUO. Thus body weight increased significantly in both groups. Since body weight, and presumably muscle mass, was different between CON and NR, blood urea nitrogen (BUN) was used to assess renal function instead of serum creatinine. BUN increased significantly after UUO in both CON and NR. There was no difference between CON and NR either before or after UUO except for body weight.

**Table 1:** Body weight, blood pressure, and blood urea nitrogen.

		CON	NR	P
Number of litters		8	7	
Body weight (g)	before UUO	171±6	134±8	0.0026
	after UUO	203±7*	161±9*	0.0021
Blood pressure				
Systolic (mmHg)	before UUO	92±4	98±4	NS
	after UUO	105±5*	112±4*	NS
Diastolic (mmHg)	before UUO	50±3	58±5	NS
	after UUO	61±4	67±7	NS
BUN (mg/dl)	before UUO	15.9±2.1	17.5±1.0	NS
	after UUO	22.7±2.8*	21.0±1.2*	NS

Means±SE. Abbreviations: CON, offspring of normally fed mothers; NR, offspring of nutrient restricted mothers; UUO, unilateral ureteral obstruction; BUN, blood urea nitrogen.

*, P<0.05 vs before UUO.

### Urine parameters

Urine from obstructed kidneys was examined for osmolality, protein-to-creatinine ratio, sodium-to-creatinine ratio, N-acetyl-β-D-glucosaminidase-to-creatinine ratio, and magnesium-to-creatinine ratio (Table 2). There was no difference in these parameters between NR and CON.

**Table 2:** Urine from the obstructed kidney.

	CON	NR
Urine chemistry (n of litters)	4	6
Osmolality (mOsm/kg)	309±3	341±16
Protein/Cr (g/g)	14.7±8.7	33.4±16.5
Na/Cr (mEq/g)	5.2±1.1	6.7±2.5
NAG/Cr (U/g)	420±93	367±156
Urine NOx (n of litters)	7	5
(nmol/mgCr)	727±164	2859±890*

CON, offspring of normally fed mothers; NR, offspring of nutrient restricted mothers; Means±SE. Abbreviations: CON, controls; NR, offspring of nutrient restricted mothers; Cr, creatnine; NAG, N-acetyl-β-D-glucosaminidase; NOx, nitrate/nitrite.

*, P<0.05 vs CON. Not all the urine parameters were measured because of insufficient amount of samples.

### Acute tubular necrosis

**Fig 1:**
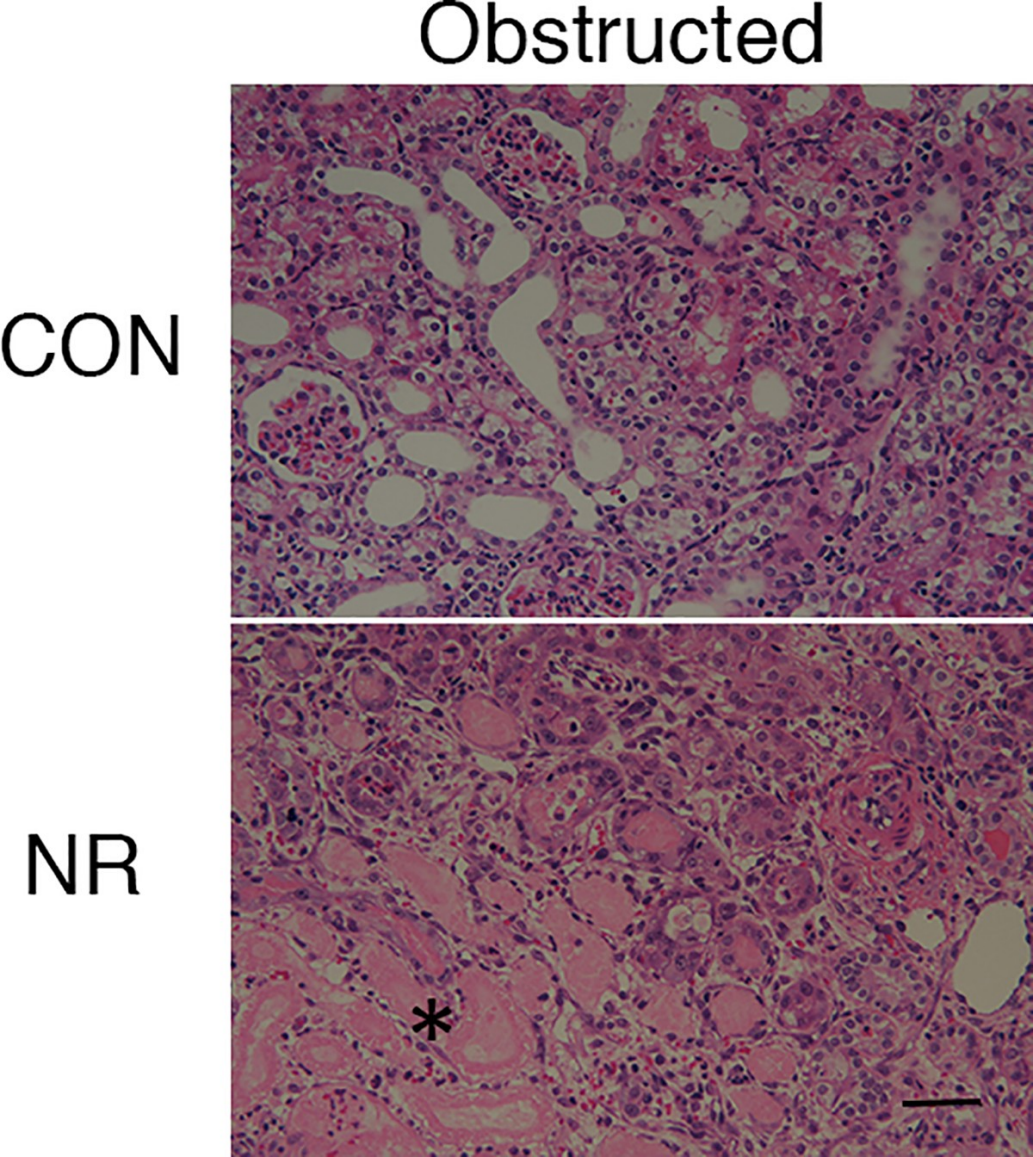
Acute tubular necrosis in the obstructed kidney of offspring from control and nutrient restricted mothers. Representative hematoxylin and eosin staining. CON, controls; NR, maternal nutrient restriction; *, necrosis of tubular cells, loss of brush border, and casts are observed. Scale bar, 50 μm.

Extensive ATN was observed in 5 out of 7 obstructed kidneys of NR (Fig 1). None of 8 obstructed kidneys of CON showed extensive ATN.

### Tubulointerstitial fibrosis

**Fig 2:**
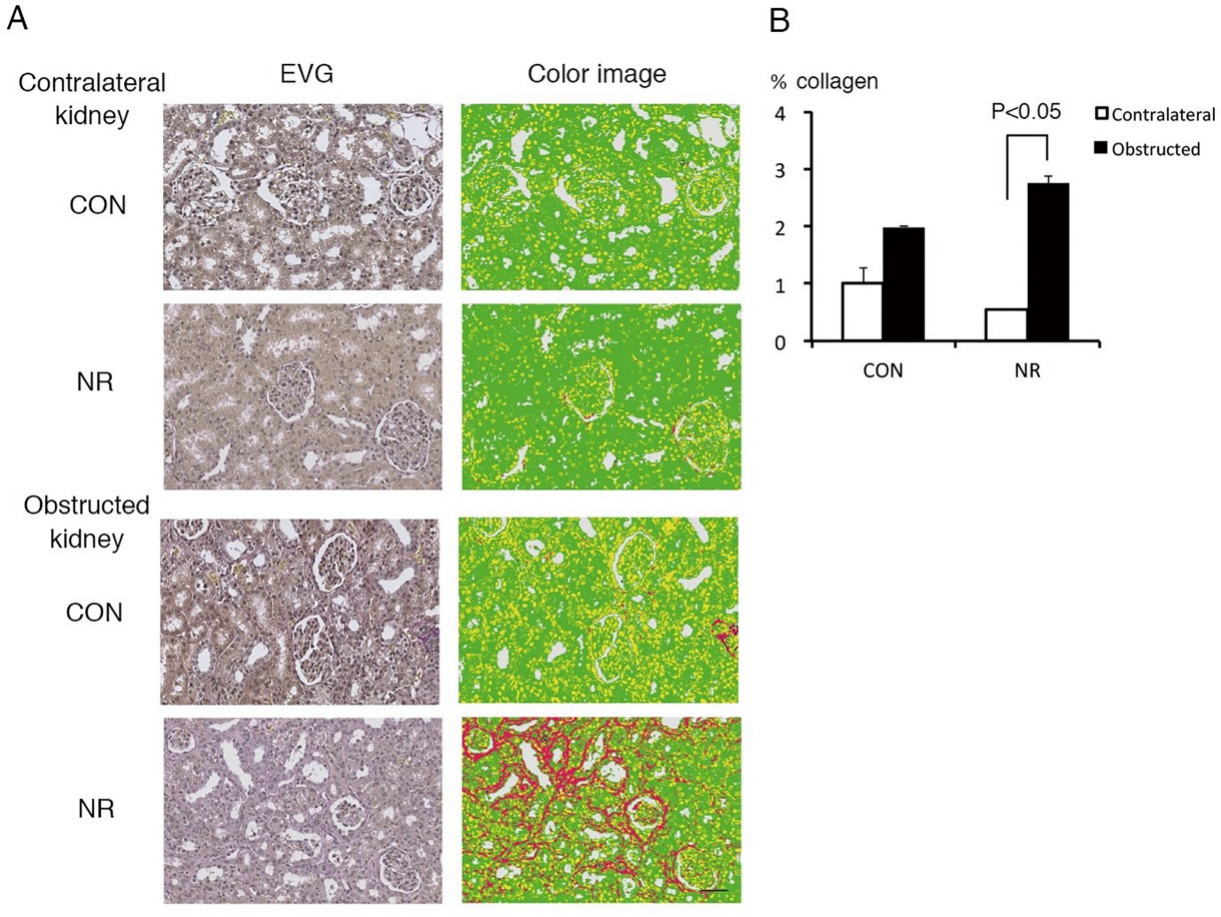
Tubulointerstitial fibrosis in the contralateral and obstructed kidney of offspring from control and nutrient restricted mothers. (A) Representative EVG staining and the color classification. CON, controls; NR, maternal nutrient restriction. After staining with EVG, WSI of each specimen was acquired. The WSI pixels were classified into collagen fibers, elastic fibers, nuclei, and cytoplasm were red, blue, yellow, and green, respectively. Scale bar, 50 μm. (B) Quantitative analysis. The area ratio of collagen fibers is the sum of pixels for collagen fibers divided by the total number of pixels of the four tissue components. n = 5 litters each. Not all the samples were examined because the variation was small easily reaching statistical significance.

Kidneys were stained with EVG and interstitial fibrosis was quantified as collagen area ratio (Fig 2A). Collagen area ratio in the contralateral kidney was low and there was no significant difference between CON and NR (Fig 2B). Collagen area ratio in the obstructed kidney was significantly larger compared with the contralateral kidney only in NR.

### Peritubular capillary density

**Fig 3:**
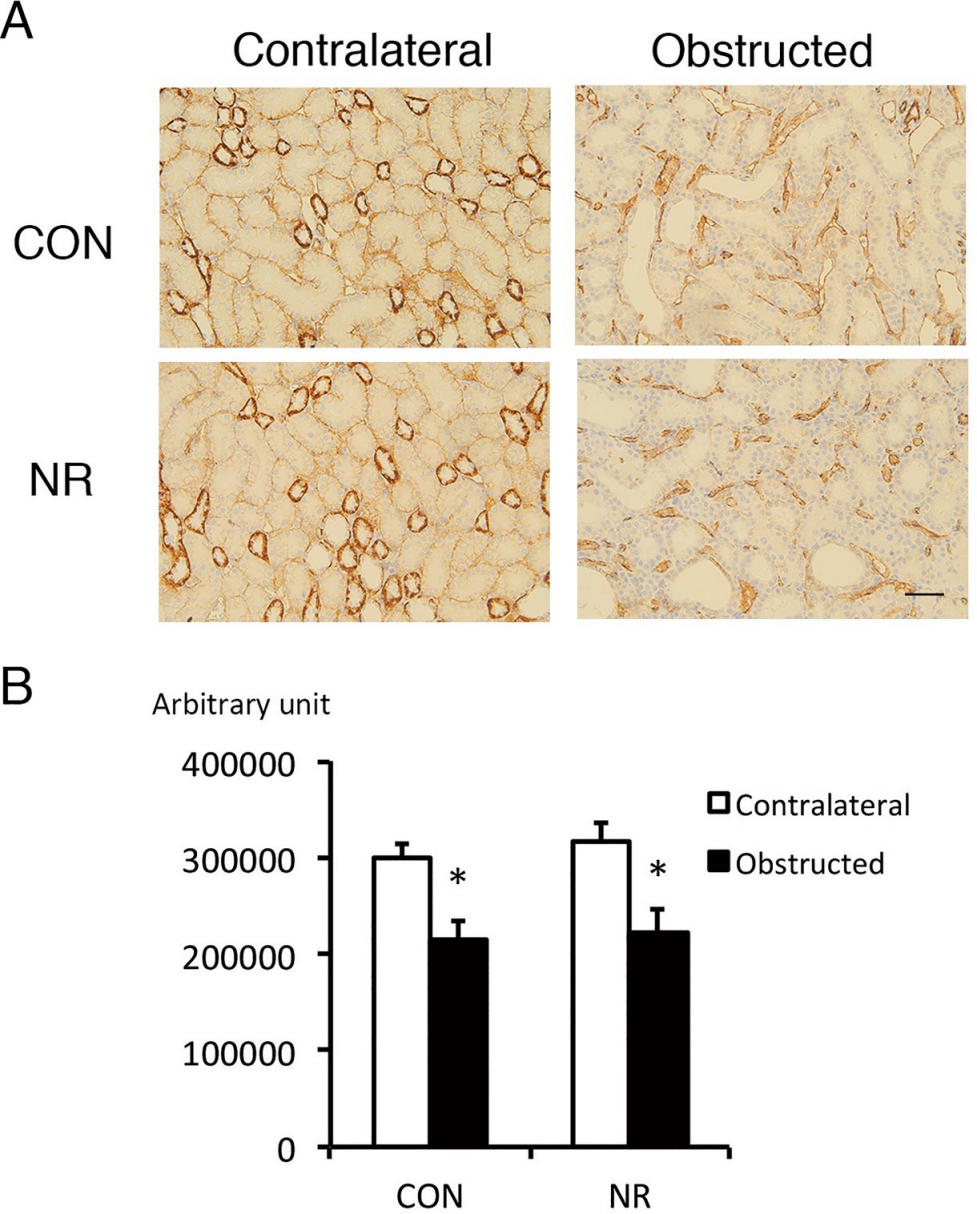
CD31-positive area in the contralateral and obstructed kidney of offspring from control and nutrient restricted mothers. (A) Representative CD31 staining. (B) Quantitative analysis. CON, controls; NR, maternal nutrient restriction. n = 4 litters each. CD31-positive area is expressed as arbitrary unit. *, P<0.05 vs contralateral kidneys. Not all the samples were examined because the variation was small easily reaching statistical significance.

We examined whether extensive ATN and interstitial fibrosis observed in NR obstructed kidneys was secondary to decreased density of peritubular capillaries, since reduced capillary density leads to renal hypoxia which characterizes UUO [17]. The area positive for the endothelial marker CD31 was significantly decreased in the obstructed kidney compared with the contralateral kidney in both CON and NR (Fig 3A). However, there was no difference between CON and NR in either obstructed or contralateral kidneys (Fig 3B).

### Urine NOx

Urine NOx, a marker of NO bioavailability, was reported to increase after UUO [18]. We examined whether there was a difference in urine NOx between NR and CON. Urine NOx from the obstructed kidney was significantly increased in NR compared with CON (Table 2).

### Endothelial nitric oxide synthase (eNOS) expression

**Fig 4:**
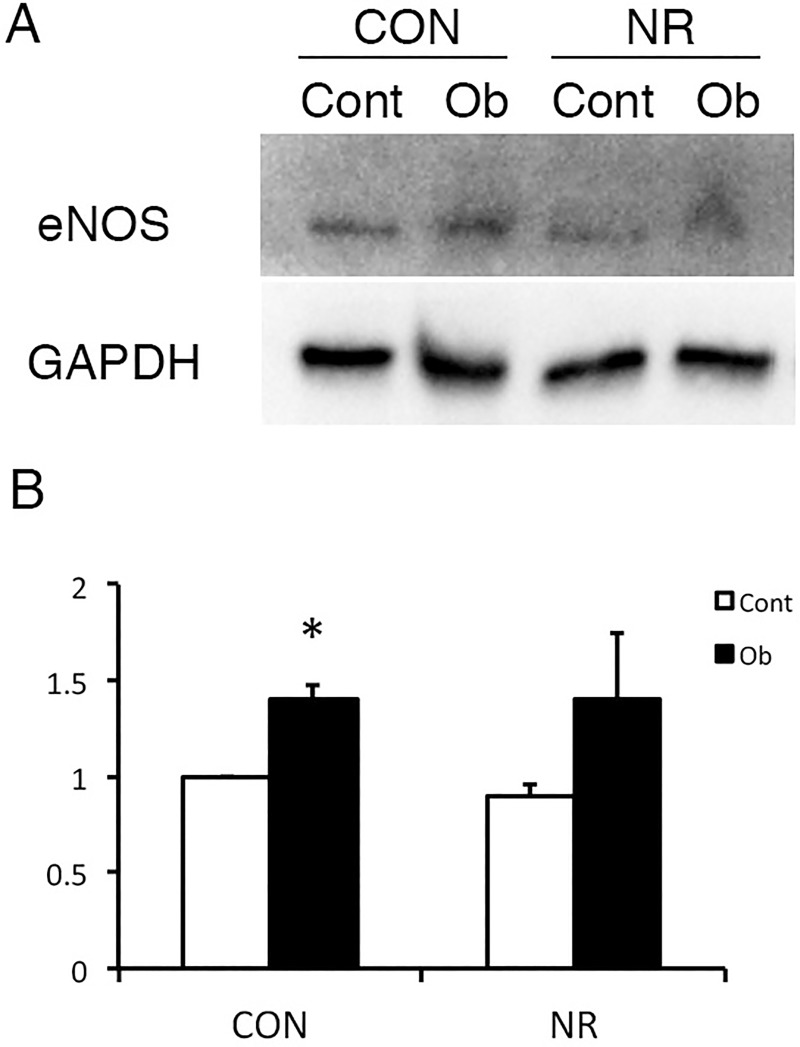
eNOS expression in the contralateral and obstructed kidney of offspring from control and nutrient restricted mothers. (A) A representative immunoblot. (B) Quantitative analysis. CON, controls; NR, maternal nutrient restriction; Cont, contralateral kidneys; Ob, obstructed kidneys; n = 4; *, P<0.05 vs Cont. Not all the samples were examined because the variation was small easily reaching statistical significance.

In association with increased NO production, increased eNOS expression has been reported in obstructed kidneys [18]. Immunoblot analysis showed that eNOS expression in the contralateral kidney was not different between CON and NR (Fig 4). The expression of eNOS was significantly greater in the obstructed kidney than the contralateral kidney in CON. In NR, however, the statistical significance was not achieved due to high variability. The eNOS level in the obstructed kidney was not different between CON and NR.

### Nitrotyrosine

**Fig 5:**
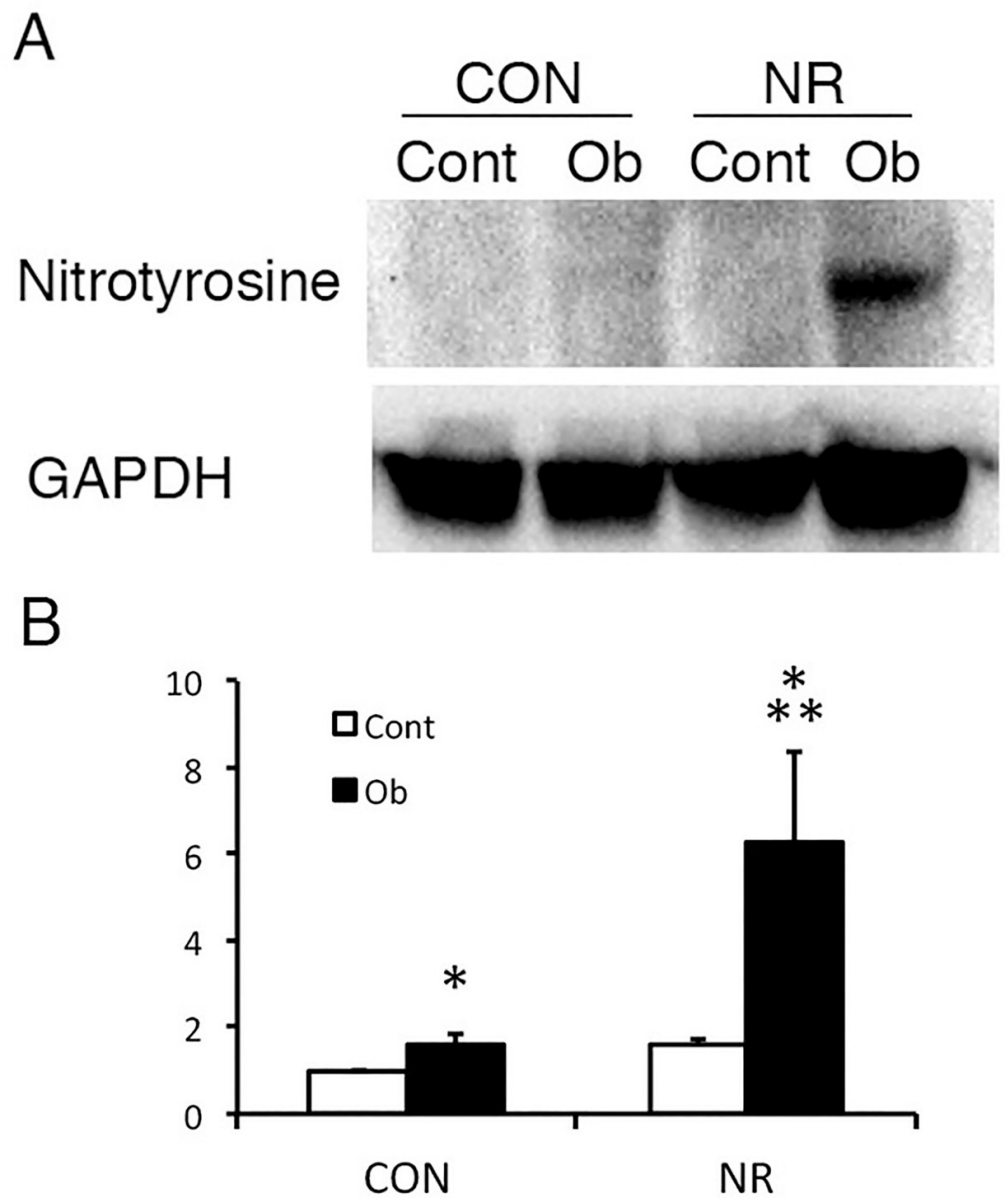
Nitrotyrosine expression in the contralateral and obstructed kidney of offspring from control and nutrient restricted mothers. (A) A representative immunoblot. (B) Quantitative analysis. CON, controls; NR, maternal nutrient restriction; Cont, contralateral kidneys; Ob, obstructed kidneys; n = 4; *, P<0.05 vs Cont; **, P<0.05 vs Ob of CON. Not all the samples were examined because the variation was small easily reaching statistical significance.

An adverse intrauterine environment is known to increase oxidative stress [19], which may aggravate injury due to obstruction resulting in more severe ATN and tubulointerstitial fibrosis. While low concentration NO alleviates renal injury in UUO, high concentration NO is cytotoxic generating peroxynitrate, a potent oxidant. We therefore examined the expression of nitrotyrosine, a marker of peroxynitrate, which was reported to be increased in the kidney of offspring of dams fed low protein diet [19]. As shown in Fig 5, the level of nitrotyrosine in the contralateral kidney was not different between CON and NR. Nitrotyrosine expression in the obstructed kidney was increased compared with the contralateral kidney, and the extent was significantly greater in NR compared with CON (Fig 5).

## Discussion

In the present study, we found that ATN and interstitial fibrosis after UUO were more severe in male offspring of nutrient-restricted rat dams. This was thought to be due to increased nitrosative stress in the obstructed kidney of NR as evidenced by increased urine NOx and nitrotyrosine expression. Oxidative stress, including nitrosative stress, is known to contribute to the pathogenesis of obstructive uropathy leading to tubular cell death and development of interstitial fibrosis [20]. Various markers of oxidative stress including superoxide anion, hydrogen peroxide, nitrotyrosine, malondialdehyde, 8-isoprostaglandin F2α, and others have been reported to be increased in UUO kidneys [20, 21]. Levels of antioxidant enzymes catalase and superoxide dismutase were decreased in the UUO kidney [21]. Mice deficient in catalase were shown to have more severe renal fibrosis after UUO indicating the importance of oxidants in the pathogenesis of UUO [22]. In support of this, exogenous antioxidants such as fluvastatin decreased the severity of fibrosis and reduced oxidative stress markers [23].

Maternal protein restriction is associated with age-associated increased oxidative stress and impairment of antioxidant defense capacity in rat islets [24]. In the same model, kidney oxidative stress was shown to contribute to the pathogenesis of hypertension [19]. In the study by Ojeda, the production of superoxide was increased in the kidney of IUGR rats both at baseline and after ischemia-reperfusion [7]. Moreover, tempol, a mimetic of superoxide dismutase, alleviated the tubular injury in IUGR rats. In the present study, the expression of nitrotyrosine in the obstructed kidney was greater in NR than CON. The increased urine NOx, a NO metabolite, also suggests increased nitrosative stress. As will be discussed later, NO has opposite cytoprotective and prooxidant actions. Prooxidant action is noted with high concentrations of NO [25]. Taken together, the more severe ATN and fibrosis in the obstructed kidney of NR may be explained by the increased nitrosative stress.

The role of NO in the kidney is complex. NO production in the obstructed kidney is known to increase, which was speculated to counterbalance angiotensin II-induced vasoconstriction [18]. The increased NO was shown to be due, in part, to increased NOS protein expression [18, 26, 27]. In UUO, eNOS, expressed in the vasculature and tubules, has been reported to be increased and is considered to protect against renal injury [18]. The loss of eNOS in peritubular capillary was associated with increased tubulointerstitial fibrosis in aging rats probably through decreased vasodilation [28]. Agents that produce NO such as L-arginine and adrenomedullin ameliorated UUO-induced fibrosis, whereas L-NAME, a NOS inhibitor, aggravated it [29, 30]. Inducible NOS (iNOS) was also shown to be increased by UUO [26]. Tubular injury after UUO was more severe in mice lacking iNOS suggesting the protective role of NO in UUO [31]. While these results suggest the beneficial action of NO, detrimental effects of NO have been reported. As stated above, NO can produce nitrosative stress. A stimulatory action of NO on collagen deposition *via* iNOS overexpression [32]and even dietary L-arginine supplementation has been reported in various organs including the kidney [33]. Animal models characterized by injurious effect of NO include lupus nephritis and others [34]. Elevated NO production was ascribed to increased iNOS activity. Genetically engineered mice with reduced tubular NO activity was shown to be protected against progressive fibrosis in folate and UUO nephropathy [35]. In that study, reduced NO activity was associated with reduced expression of collagen. Thus NO could be injurious in the UUO model depending on the setting.

Evidence suggests that cytotoxic effects of NO is attributed to peroxynitrite which is produced from the reaction between NO and superoxide anion [36]. Peroxynitrite then oxidizes lipids and nitrates tyrosine residues of proteins forming nitrotyrosine. Thus nitrotyrosine is considered to be a marker of perooxynitrite formation as well as NO production. In the present study, nitrotyrosine was upregulated in the obstructed kidney of NR compared with CON indicating the increased formation of peroxynitrite. One of the mechanisms of action of peroxynitrite is cell signaling. Of particular relevance to UUO is the activation of ERK by peroxynitrite [36]. Thus ERK has been shown to be involved in fibrosis, apoptosis, and TGF-ß1 production induced by kidney obstruction [23, 37].

In animal models of IUGR, eNOS in aorta and pulmonary artery endothelial cells was reported to be decreased [38, 39]. In our study, however, eNOS expression in NR was not different from that in CON in either the contralateral or obstructed kidney. In contrast to eNOS, iNOS expression was reported to be elevated in the kidney of IUGR rabbits induced by uteroplacental ligation, which was associated with increased reactive oxygen species and nitrotyrosine [40]. Increase in iNOS, therefore, may be the cause of the increased NO and subsequent formation of nitrotyrosine in the obstructed kidney of NR.

Capillary rarefaction is known to occur in various organs by an adverse intrauterine environment [41]. In the kidney, we recently reported peritubular capillary rarefaction in humans born with extremely low birth weight and IUGR [3]. Reduced peritubular capillaries, a crucial factor in the progression of fibrosis *via* aggravation of hypoxia, have also been reported in adult sheep exposed to maternal protein restriction [42]. In UUO, capillary rarefaction has been observed and associated with tubulointerstitial fibrosis. In the present study also, capillary density was decreased in the obstructed kidney compared with the contralateral kidney. There was, however, no difference between CON and NR in either contralateral or obstructed kidneys.

While more severe tubulointerstitial damage in IUGR models may have been ascribed to greater glomerular injury, we showed that maternal nutrient restriction has a direct effect on the tubulointerstitium since one-week UUO does not induce glomerulosclerosis. Of note, our previous study in extremely low birth weight survivors found that the frequency of elevated urine ß2 microglobulin, a marker of tubulointerstitial damage, increased with age in no association with low glomerular filtration rate [2]. It is possible that urine ß2 microglobulin or urine NOx may prove to be a marker for intrauterine stress. Also, other animal models with direct injury to the tubulointerstitium such as folic acid or cyclosporine A nephropathy may be helpful to confirm the effect of programming in renal fibrosis. The mechanisms for the increased oxidative/nitrosative stress in the kidney programmed by adverse intrauterine events need to be investigated. Epigenetic modifications are likely to play a role, which may give an opportunity for intervention.

Limitation of the present study is that we could not demonstrate the difference in blood pressure or renal function between CON and NR both before and after UUO. A more precise technique of measuring blood pressure, with telemetry in conscious rats, and or a higher number of litters may reveal the difference. The same applies to glomerular filtration rate. Also, we could not demonstrate the difference in other oxidative stress markers even if we tried several markers including urine 8-iso-prostaglandin F2α and renal malondealdehyde expression.

## Conclusions

Maternal nutrient restriction aggravates tubular necrosis and interstitial fibrosis after UUO in the offspring. The cause seems to be increased oxidative stress probably primed by the adverse intrauterine environment.

## Supporting information

S1 File NC3Rs ARRIVE guidelines checklist. (PDF)

S1 Fig Original raw image for Fig 1 CON. (JPG)

S2 Fig Original raw image for Fig 1 NR. (JPG)

S3 Fig Original raw image for Fig 2 CON contralateral. (JPG)

S4 Fig Original raw image for Fig 2 CON obstructed. (JPG)

S5 Fig Original raw image for Fig 2 NR contralateral. (JPG)

S6 Fig Original raw image for Fig 2 NR obstructed. (JPG)

S7 Fig Original raw image for Fig 3 CON CON. (JPG)

S8 Fig Original raw image for Fig 3 NR CON. (JPG)

S9 Fig Original raw image for Fig 3 CON Ob. (JPG)

S10 Fig Original raw image for Fig 3 NR Ob. (JPG)

S11 Fig Original raw image for Fig 4 eNOS. Left half is the used image.
(TIF)

S12 Fig Original raw image for Fig 4 GAPDH. Left half is the used image.
(TIF)

S13 Fig Original raw image for Fig 5 nitrotyrosine. Upper half is the used image.
(TIF)

S14 Fig Original raw image for Fig 5 GAPDH. Right lower is the used image.
(TIF)
