# Supplementary figures and images for: Maternal undernutrition aggravates renal tubular necrosis and interstitial fibrosis after unilateral ureteral obstruction in male rat offspring
Source: PLoS One. 2019 Sep 3;14(9):e0221686. doi: 10.1371/journal.pone.0221686 (PMC6719870; doi:10.1371/journal.pone.0221686)

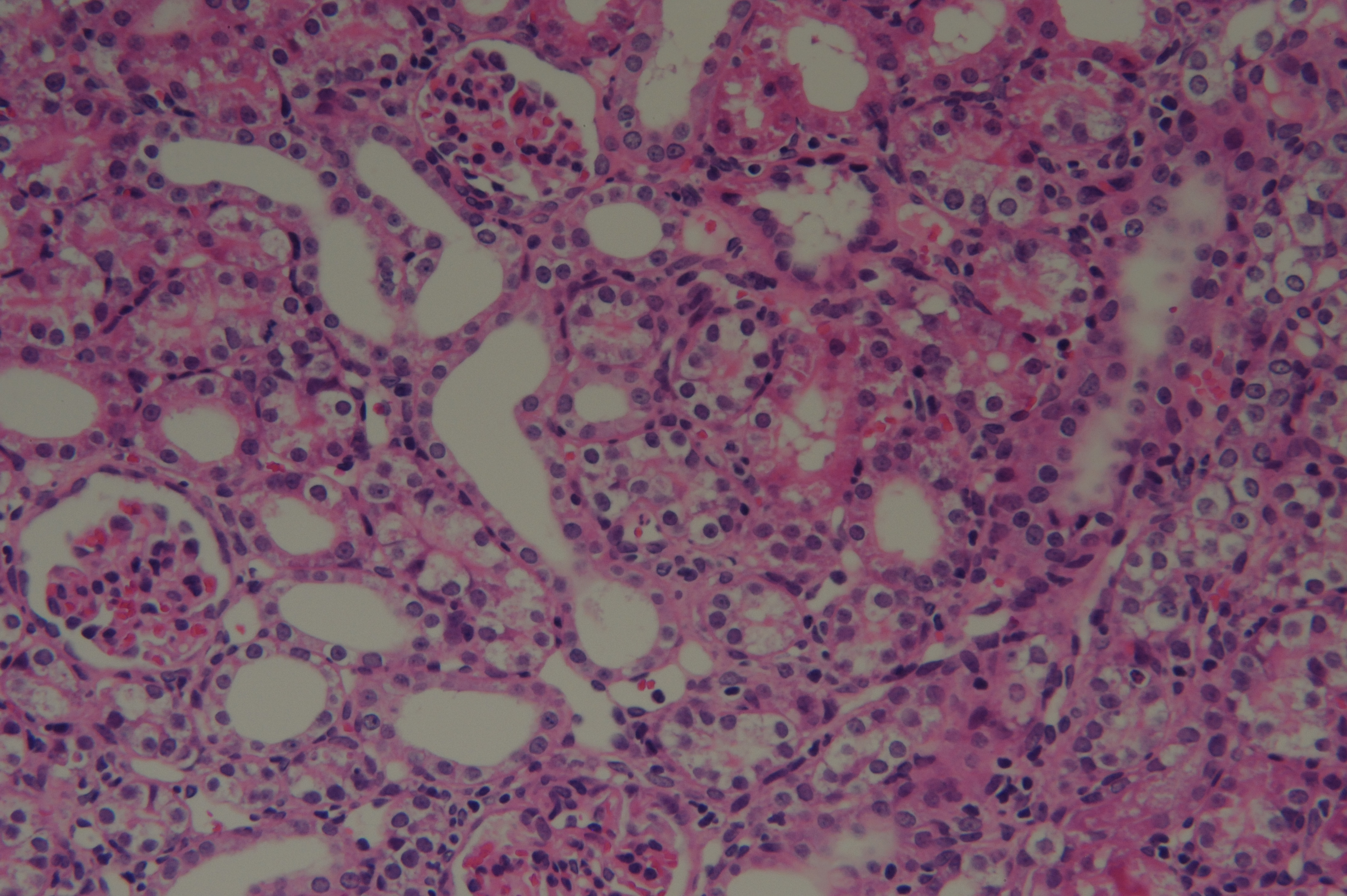

Supplement: S1 Fig — (JPG) [file pone.0221686.s002.jpg]

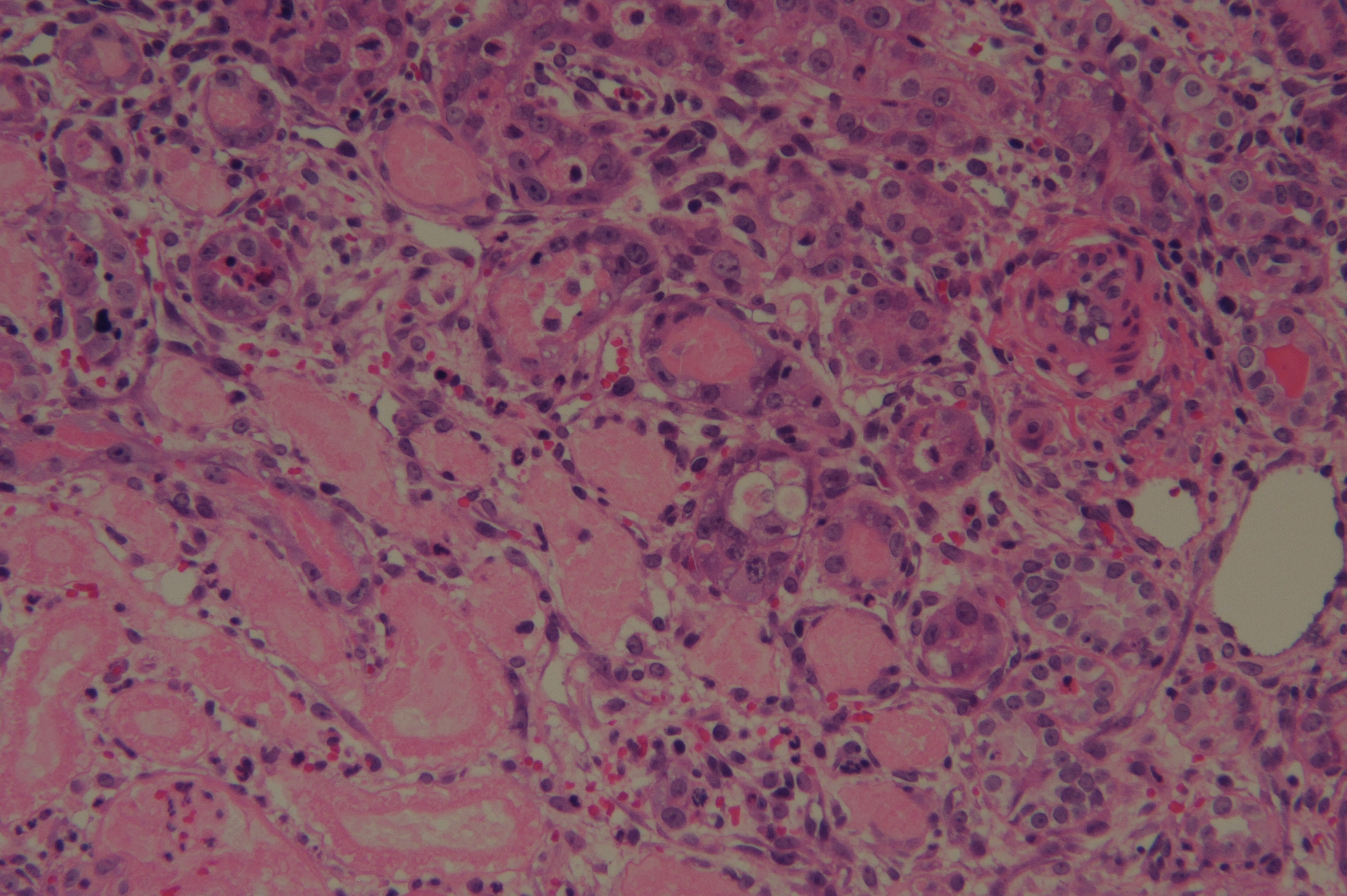

Supplement: S2 Fig — (JPG) [file pone.0221686.s003.jpg]

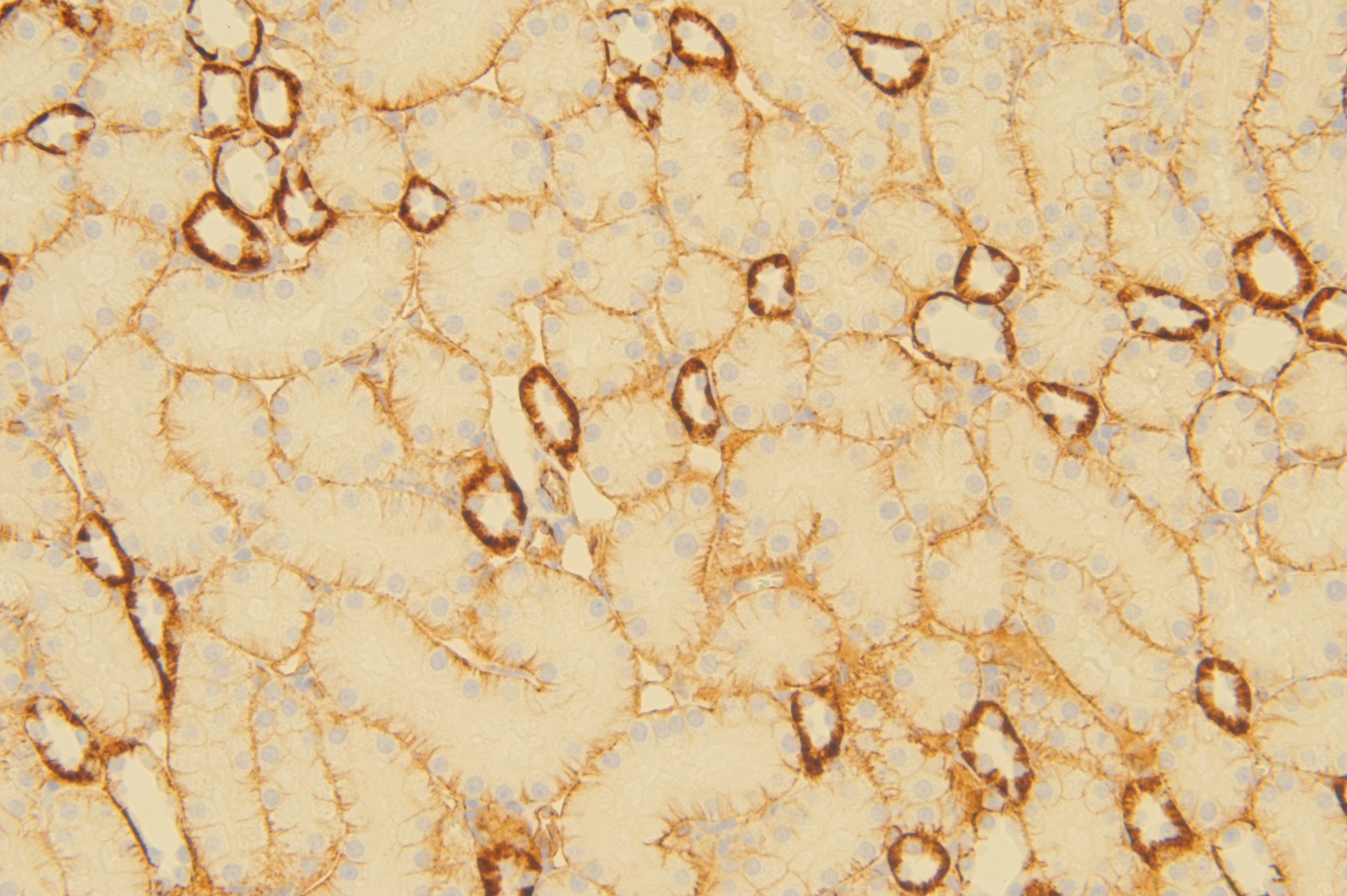

Supplement: S7 Fig — (JPG) [file pone.0221686.s008.jpg]

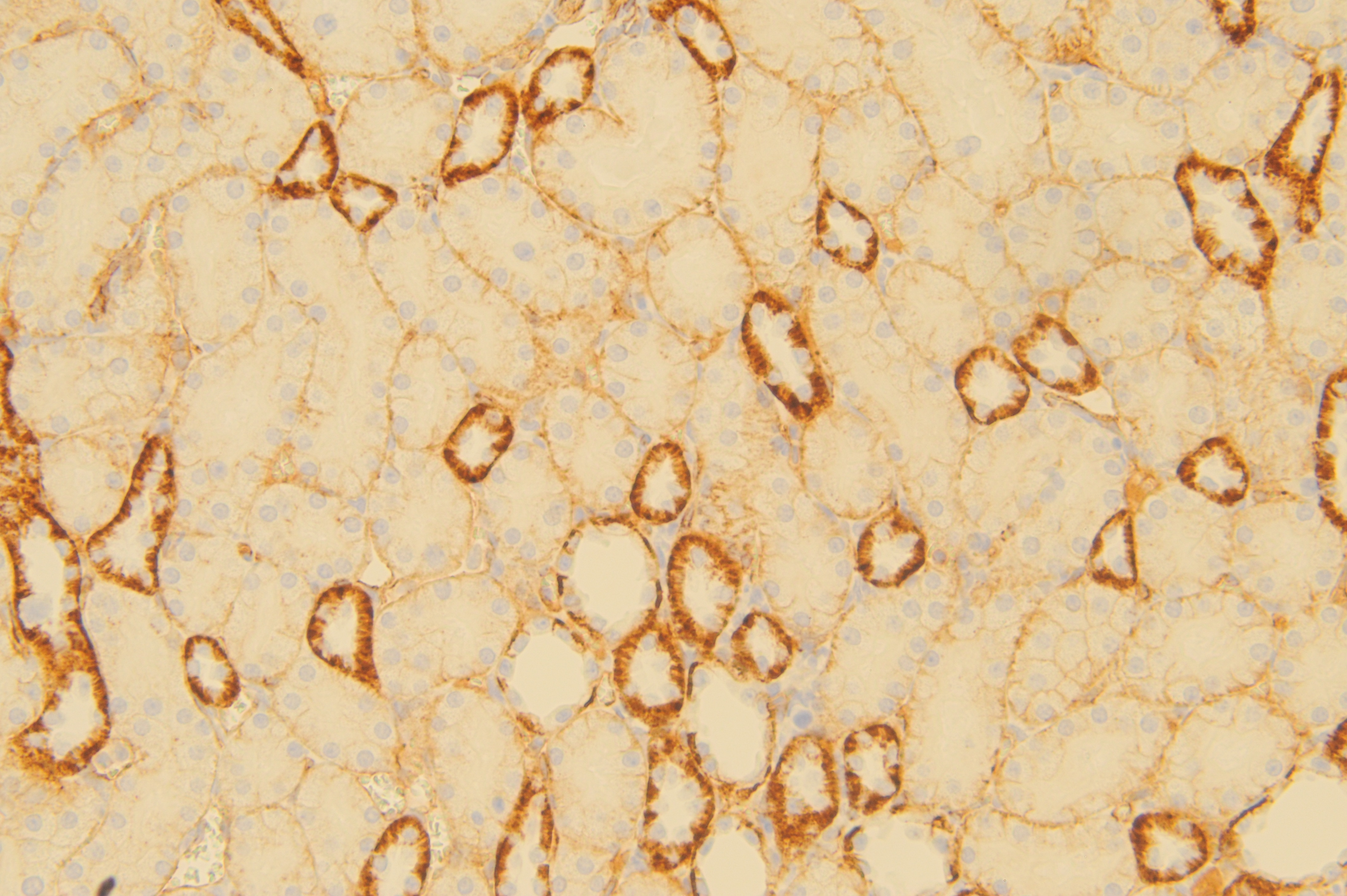

Supplement: S8 Fig — (JPG) [file pone.0221686.s009.jpg]

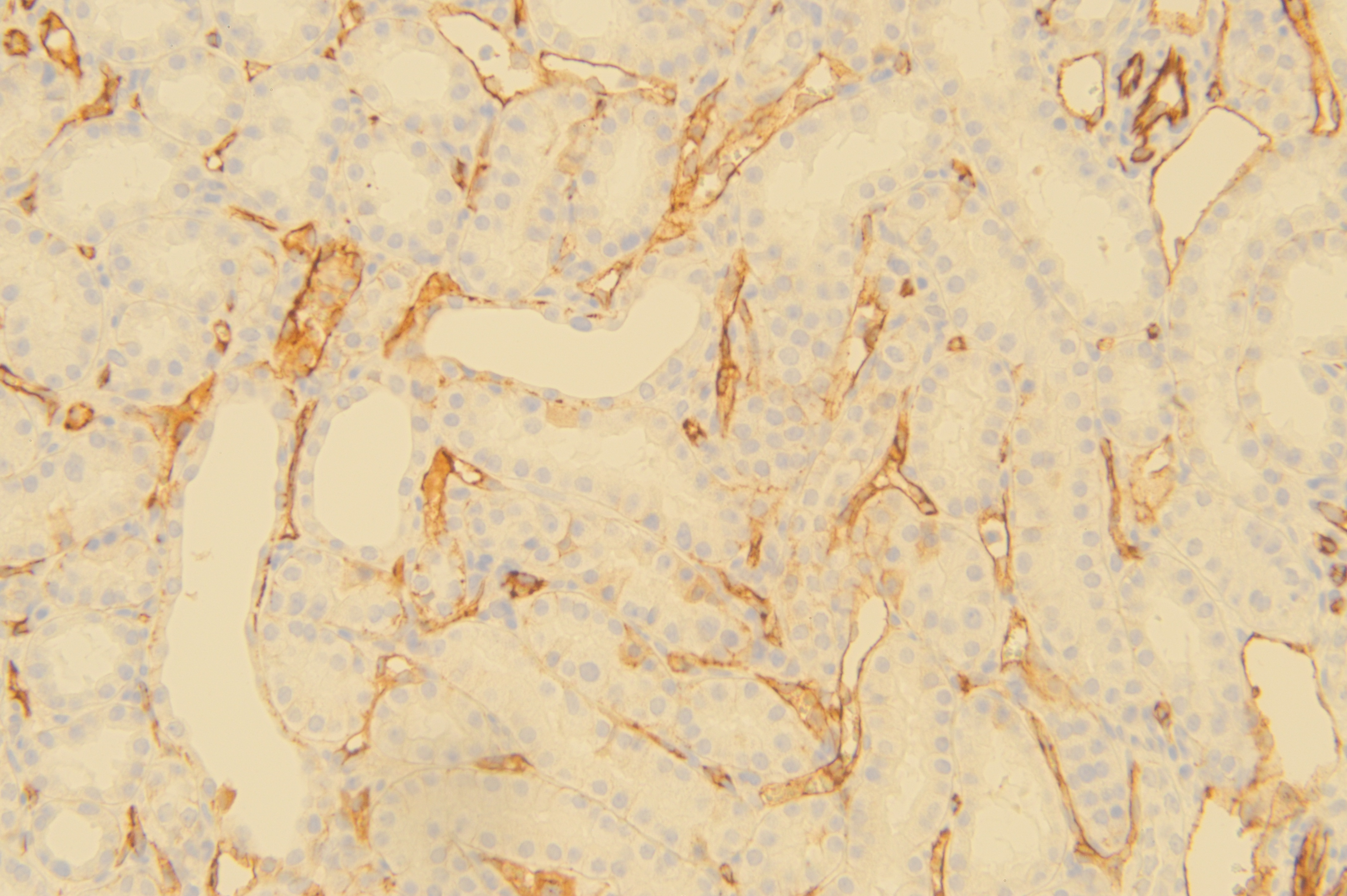

Supplement: S9 Fig — (JPG) [file pone.0221686.s010.jpg]

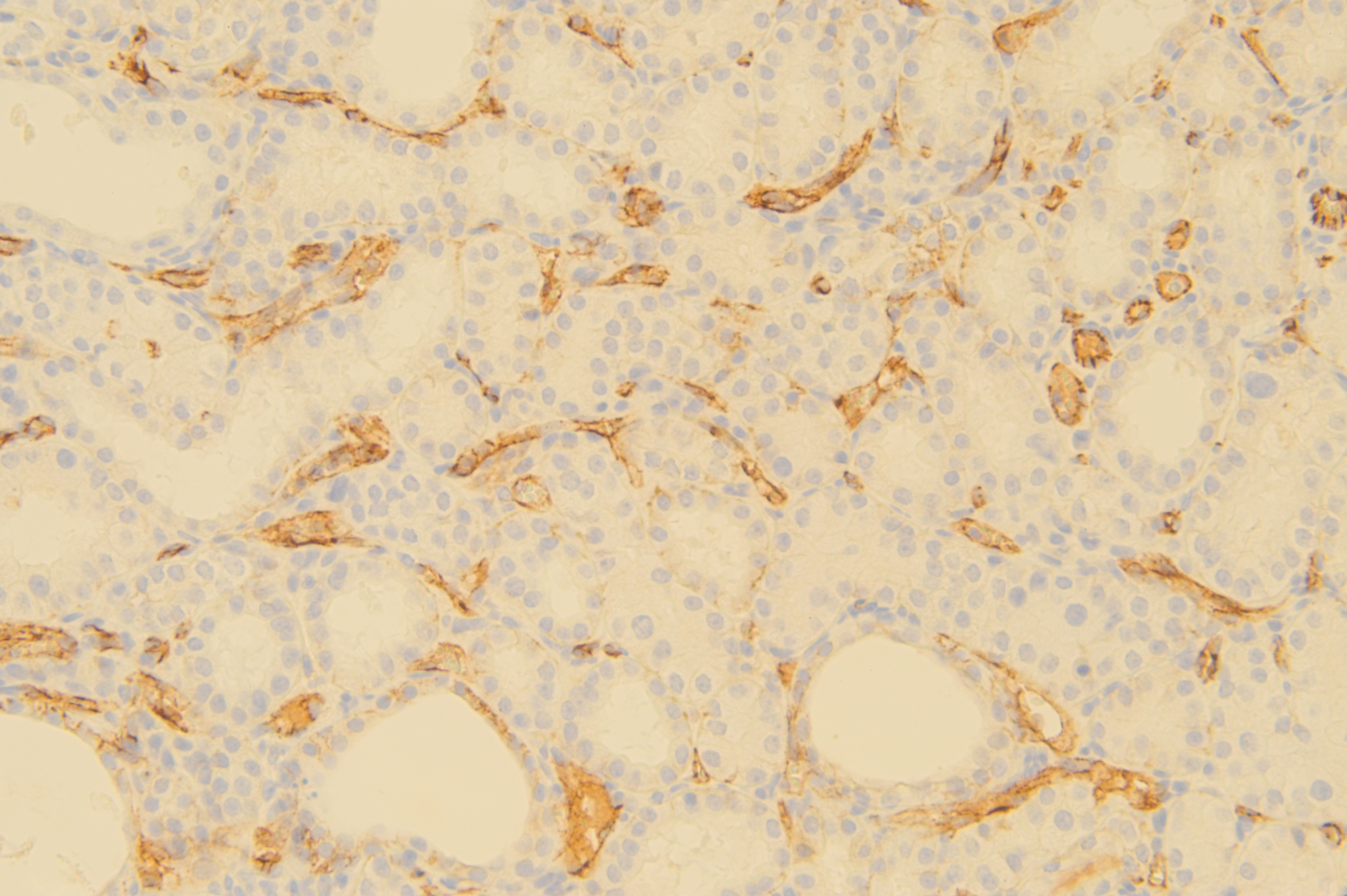

Supplement: S10 Fig — (JPG) [file pone.0221686.s011.jpg]

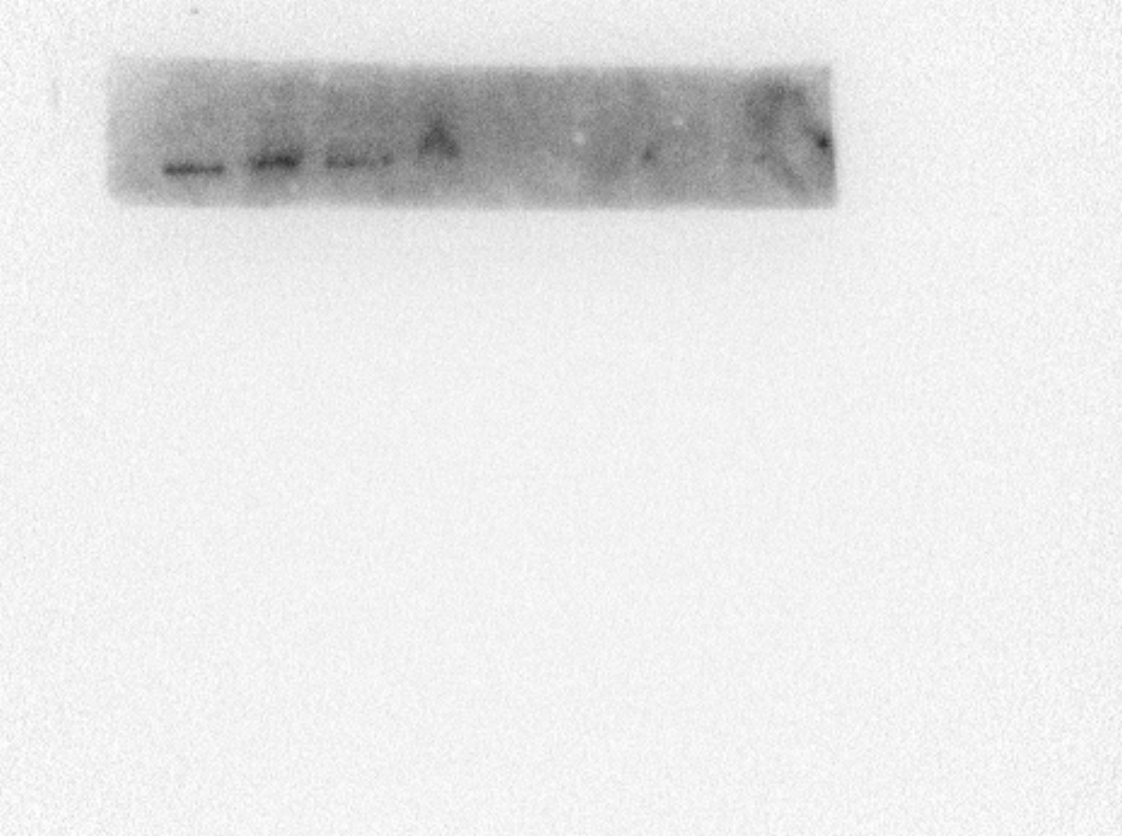

Supplement: S11 Fig — Left half is the used image. (TIF) [file pone.0221686.s012.tif]

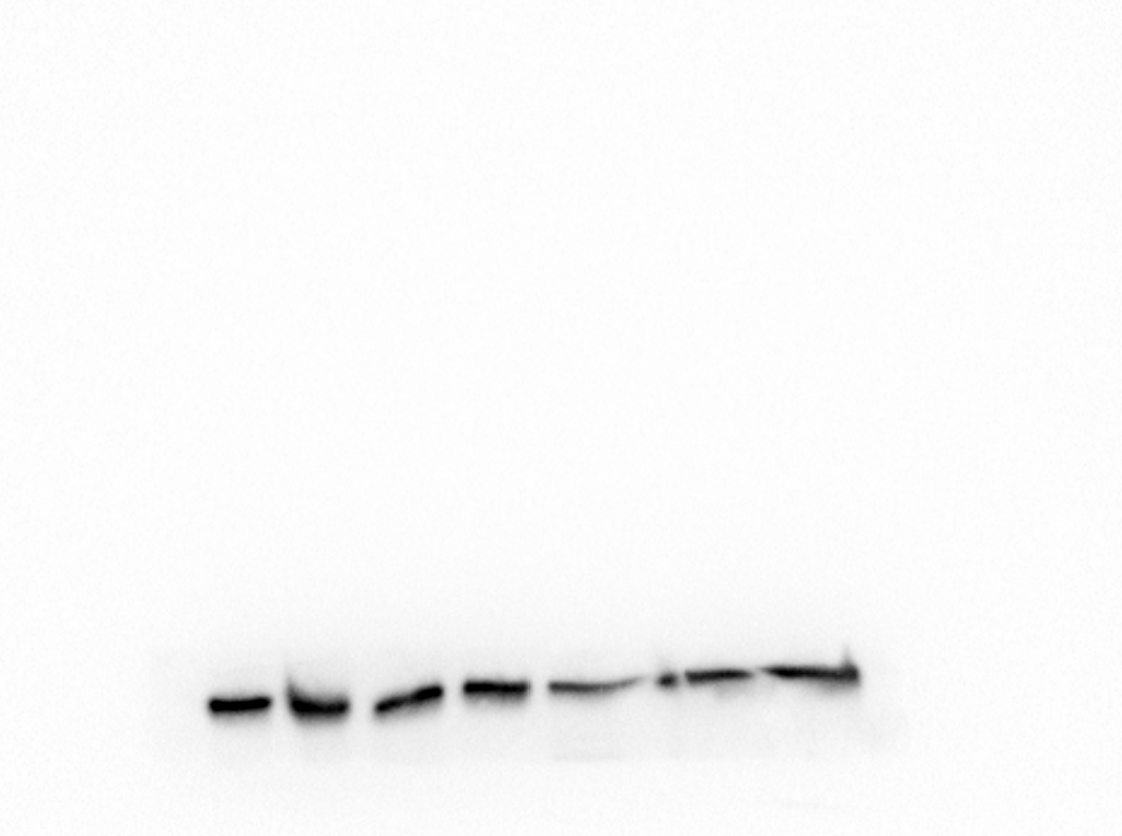

Supplement: S12 Fig — Left half is the used image. (TIF) [file pone.0221686.s013.tif]

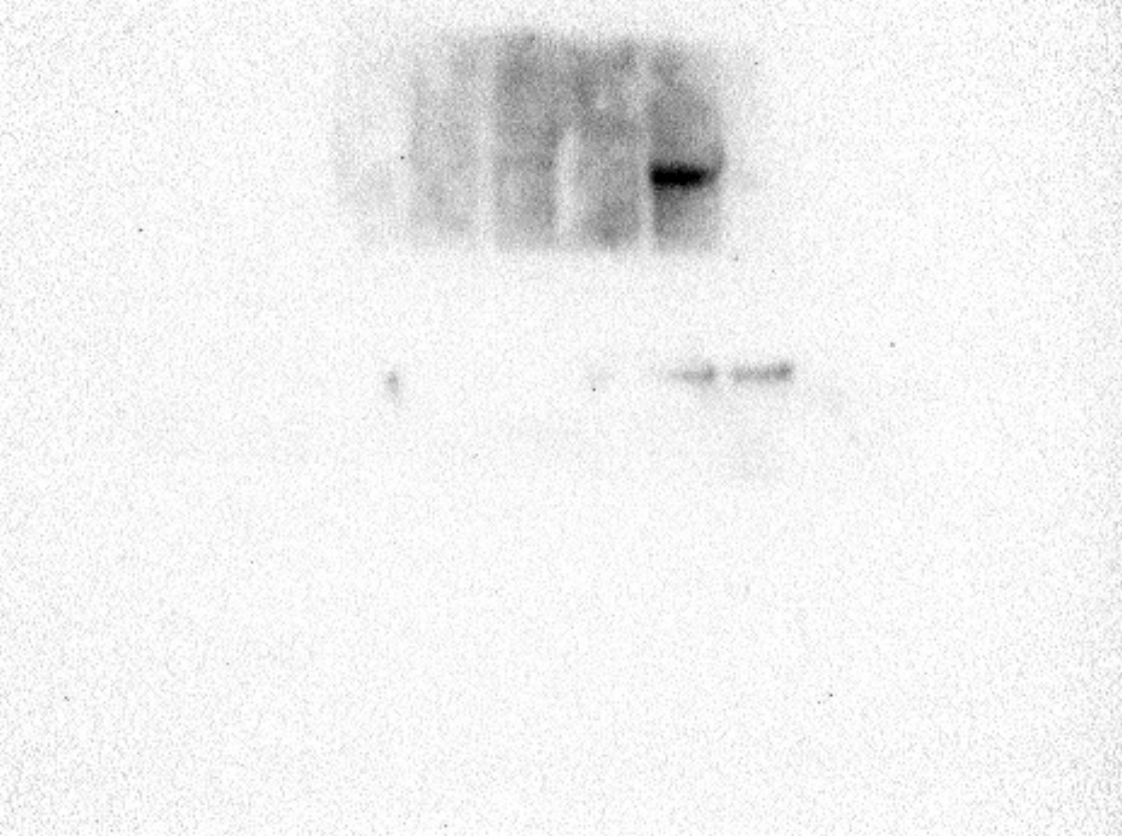

Supplement: S13 Fig — Upper half is the used image. (TIF) [file pone.0221686.s014.tif]

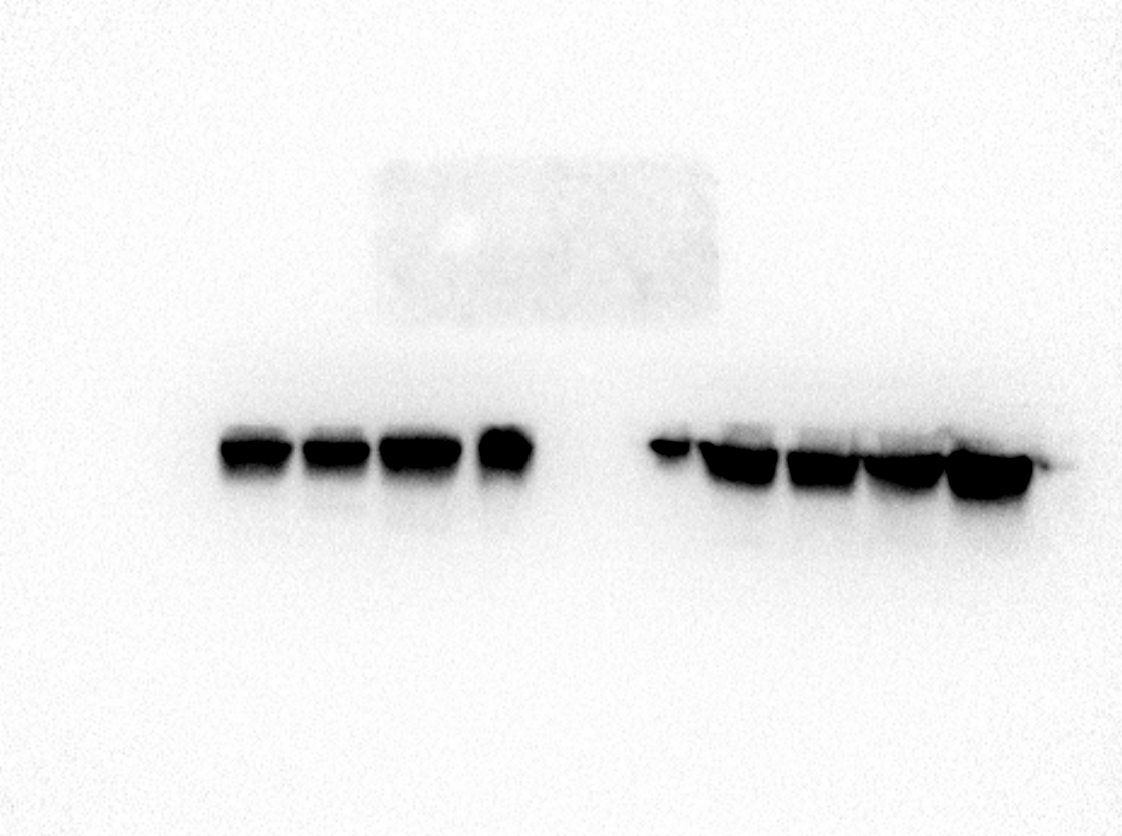

Supplement: S14 Fig — Right lower is the used image. (TIF) [file pone.0221686.s015.tif]
